# Severity of Bovine Tuberculosis Is Associated with Co-Infection with Common Pathogens in Wild Boar

**DOI:** 10.1371/journal.pone.0110123

**Published:** 2014-10-28

**Authors:** David Risco, Emmanuel Serrano, Pedro Fernández-Llario, Jesús M. Cuesta, Pilar Gonçalves, Waldo L. García-Jiménez, Remigio Martínez, Rosario Cerrato, Roser Velarde, Luis Gómez, Joaquím Segalés, Javier Hermoso de Mendoza

**Affiliations:** 1 Red de Grupos de Investigación en Recursos Faunísticos, Facultad de Veterinaria, Universidad de Extremadura, Cáceres, Spain; 2 Centre for Environmental and Marine Studies (CESAM), Departamento de Biología, Universidade de Aveiro, Aveiro, Portugal; 3 Servei d'Ecopatologia de Fauna Salvatge (SEFaS), Departament de Medicina i Cirurgia Animals, Universitat Autònoma de Barcelona, Bellaterra, Spain; 4 Centre de Recerca en Sanitat Animal (CReSA), Universitat Autònoma de Barcelona – l″Institut de Recerca i Tecnologia Agroalimentàries, Bellaterra, Spain; 5 Departament de Sanitat i Anatomia Animals, Universitat Autònoma de Barcelona, Bellaterra, Spain; INIAV, I.P.- National Institute of Agriculture and Veterinary Research, Portugal

## Abstract

Co-infections with parasites or viruses drive tuberculosis dynamics in humans, but little is known about their effects in other non-human hosts. This work aims to investigate the relationship between *Mycobacterium bovis* infection and other pathogens in wild boar (*Sus scrofa*), a recognized reservoir of bovine tuberculosis (bTB) in Mediterranean ecosystems. For this purpose, it has been assessed whether contacts with common concomitant pathogens are associated with the development of severe bTB lesions in 165 wild boar from mid-western Spain. The presence of bTB lesions affecting only one anatomic location (cervical lymph nodes), or more severe patterns affecting more than one location (mainly cervical lymph nodes and lungs), was assessed in infected animals. In addition, the existence of contacts with other pathogens such as porcine circovirus type 2 (PCV2), Aujeszky's disease virus (ADV), swine influenza virus, porcine reproductive and respiratory syndrome virus, *Mycoplasma hyopneumoniae*, *Actinobacillus pleuropneumoniae, Haemophilus parasuis* and *Metastrongylus* spp, was evaluated by means of serological, microbiological and parasitological techniques. The existence of contacts with a structured community of pathogens in wild boar infected by *M. bovis* was statistically investigated by null models. Association between this community of pathogens and bTB severity was examined using a Partial Least Squares regression approach. Results showed that adult wild boar infected by *M. bovis* had contacted with some specific, non-random pathogen combinations. Contact with PCV2, ADV and infection by *Metastrongylus* spp, was positively correlated to tuberculosis severity. Therefore, measures against these concomitant pathogens such as vaccination or deworming, might be useful in tuberculosis control programmes in the wild boar. However, given the unexpected consequences of altering any community of organisms, further research should evaluate the impact of such measures under controlled conditions. Furthermore, more research including other important pathogens, such as gastro-intestinal nematodes, will be necessary to complete this picture.

## Introduction

Co-infections (i.e., the simultaneous infection of a host by two or more pathogens) are ubiquitous in nature but most research on relevant diseases largely relies on a “one-disease-one-pathogen” perspective. From the point of view of community ecology, a host can be considered a complex ecosystem composed of parasites that directly or indirectly interact among themselves and with their own environment, the host [1]. This holistic perspective considers co-infections as specific cases of competition [2] that regulate parasite populations within the host, either protecting (see Reich et al. 2013 for a case of cross-immunity [3]) or driving infection risk [4]. Interestingly, such interactions are possible between microparasites (virus, bacteria, fungi or protozoa) and macroparasites (helminths and arthropods) inhabiting different organs (i.e., arthropods infecting nasal cavities drive gastrointestinal nematode fitness [5]), and, thus, predicting the outcome of co-infection is a complex task.

Among all possible interactions, bacteria-helminth co-infections are one of the most studied models for exploring how co-infection drives disease dynamics and severity. Helminths mostly induce cytokines associated with a T-helper cell type 2 (Th2) immune response, which simultaneously tends to down-regulate T-helper cell type 1 (Th1) cytokines involved in intracellular microparasite control [6]. The consequences of this antagonism in immune mechanisms, in terms of changes in dynamics of bacteria or helminth populations, are difficult to predict [7]. A well-known example of this complexity is the bacteria-helminth co-infection in wild rabbits (*Oryctolagus cuniculus*). In this host-parasite model, respiratory infection by *Bordetella bronchiseptica* facilitates secondary helminth (*Graphidium strigosum*) infections that, at the same time, collaborate in maintaining the bacterial persistence in the upper respiratory tract [8]. *B. bronchiseptica* infection does not only facilitate *Heligmosomoides polygyrus* reproduction, another gastrointestinal nematode [9], but also accelerates the expulsion of a third worm species (*Trichostrongylus retortaeformis*) from the small intestine [10].

Shifts in disease severity are also common in a broad range of co-infected hosts. Concomitant infections often exacerbate the effects of single infections, independently of the diversity in parasite groups involved in the infection process. For example, in human tuberculosis (caused by *Mycobacterium tuberculosis*), susceptibility and severity are shaped by co-infection with different types of pathogens. Since the first report describing severe pulmonary *Mycobacterium* spp-helminth co-infected patients in the mid 1940's [11], the number of works describing changes in tuberculosis pathology due to micro- or macroparasite co-infection has grown year after year [12]. Viruses also seem to shape tuberculosis dynamics, and HIV-*Mycobacterium* spp co-infection in humans is one of the best-known examples. In fact, the HIV infection is considered one of the main risk factors predisposing patients to tuberculosis as well as the progression to active disease, increasing the risk of latent tuberculosis reactivation 20-fold [13].

Wildlife is an excellent model for exploring whether co-infection drives infectiousness of major diseases since they are almost always co-infected by several pathogens [14]. Bovine tuberculosis (bTB) due to *Mycobacterium bovis* is one of them and it is present in a broad range of wild hosts across different geographic regions [15]. Cervids in North America, badgers (*Meles meles*) in Great Britain, brush tailed possums (*Trichosurus vulpecula*) in New Zealand, buffalo (*Syncerus cafer*) in South Africa and wild boar (*Sus scrofa*) in the Iberian Peninsula are common reservoirs of this infectious disease [16]. The effects of co-infection have been described in some of these wild models. Thus, in the African buffalo, nematode infection not only is likely to increase bTB susceptibility [17], but also to accelerate mortality due to body condition impairment in co-infected individuals [18]. A positive relationship between porcine circovirus type 2 (PCV2) and bTB prevalences has also recently been observed in wild boar populations from mid-western Spain [19]; however little is known about the role of other common pathogens in wild boar in bTB dynamics.

The aim of this work was to assess whether bTB severity in wild boar from mid-western Spain is associated with the contact with a selected group of common pathogens. Evidence of infection by means of serology and/or pathogen detection was carried out for viruses (PCV2, Aujeszky's disease virus [ADV], porcine reproductive and respiratory syndrome virus [PRRSV] and swine influenza virus [SIV]), bacteria (*Mycoplasma hyopneumoniae*, *Haemophilus parasuis* and *Actinobacillus pleuropneumoniae*) and a nematode (*Metastrongylus* spp) in 165 wild boar. Two hypotheses were tested. The first investigated whether contact with a selected group of pathogens in *Mycobacterium* spp infected wild boar occurred by chance or, on the contrary, was due to a structured community of pathogens (hypothesis *i*). The second was supported by links between tuberculosis severity and concomitant viral and nematode infections observed in both human [12], [13], [20] and animal hosts [18] and explored whether disease severity in wild boar is associated to particular pathogen assemblages (hypothesis *ii*).

## Materials and Methods

### Ethics Statement

Animals included in this study belonged to private estates and were studied and sampled with the permission of their respective game managers after being hunted in commercial or sportive game activities. Since this study was carried out in private lands, no specific permissions or government approval, were required. Wild boar were hunted during game activities called “monterias” that took place according to legal guidelines. Thus, the animals were not killed specifically for this study. The development of this study did not involve any endangered or protected species in the studied area (mid-western Spain). This study did not need to be approved by any animal ethics committee since animals were not killed for scientific purposes.

### Study site

**Figure 1:**
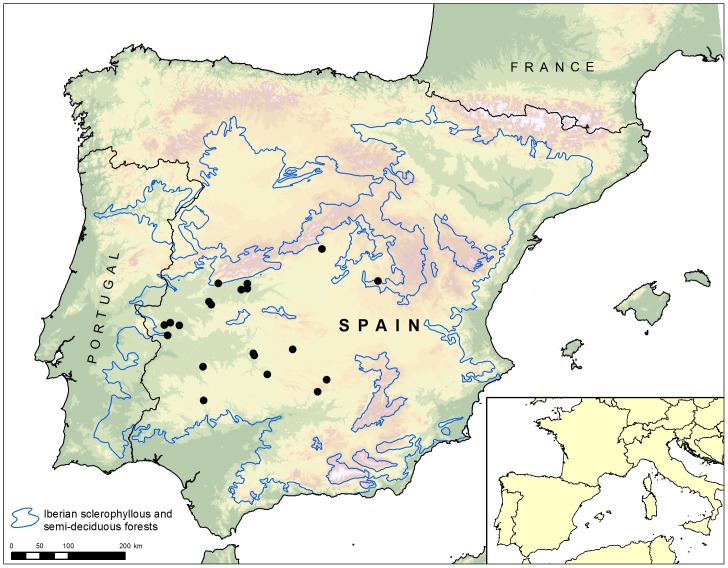
The study area is located in mid-western Spain where wild boar is the most important reservoir of bovine tuberculosis.

This study was carried out on 20 wild boar game estates in mid-western Spain (Figure 1). In this area the average annual precipitation reaches 623 mm and is concentrated from November to April. The mean annual temperature averages 17.7°C, January being the coldest and July the warmest month of the year. The vegetation is typical of Mediterranean forest, characterized by abundant *Quercus ilex* and *Q. suber* trees with understoreys dominated by *Q. coccifera*, *Cistus ladanifer* and *Erica arborea*. Wild boar density in the studied area ranged between 6.5 and 30 wild boar/hectare [21]. In all game estates included in this work, wild boar shared habitat with red deer (*Cervus elaphus*) and, in some cases, with fallow deer (*Dama dama*), roe deer (*Capreolus capreolus*) or extensive herds of cattle.

### Sampling procedures

A total of 165 hunter harvested wild boar were collected between October 2011 and February 2013. The sex and age of these animals were determined on the basis of the observation of their sexual organs and the eruption of dentition pattern, respectively [22]. Necropsy examination of all animals was performed in the field with detailed macroscopic inspection, in order to assess the presence of bTB-like visible lesions affecting lymph nodes (submandibular, retropharyngeal, mediastinal and mesenteric lymph nodes), and thoracic and abdominal organs. Submandibular and/or retropharyngeal lymph nodes, lungs and blood samples collected from the heart or thoracic cavity, were recovered and stored at 4°C until they were processed within the following 24 hours. Blood samples were centrifuged at 3000 rpm for 10 minutes and extracted serum was stored at −20°C until analysis. Field necropsies were carried out by the same person (DR), following the same protocol and criteria to evaluate the presence of bTB-like visible lesions.

### bTB diagnosis

Diagnosis of bTB in wild boar was carried out based on the isolation of *M. bovis* as well as on the presence of microscopic granulomatous bTB lesions. Since the combination of microbiological culture and histopathology increases the sensitivity and specificity of bTB wild boar surveys [23], animals that were positive for both, or at least according to one of these diagnostic techniques, were considered to be positive for bTB.

To detect the presence of *M. bovis*, microbiological cultures from intact (not handled or cut) submandibular or retropharyngeal lymph nodes and from a piece of caudal lung lobes (both with gross bTB-like lesions when possible) of each animal were carried out. For bacterial culture, tissue samples were sectioned and dissected, trimming the fat and connective tissue, using sterile scissors and forceps for each individual sample. Two grams of tissue were homogenized in 10 ml of sterile water with 0.2% albumin (Albumin from bovine serum Sigma, St Louis, MO, USA) for 4 minutes in a mechanic homogenizer (Smasher; AES Laboratories, Montreal, QC, Canada). The homogenized material was then decontaminated by the hexadecyl pyridinium chloride method [24]. Finally, two Lowenstein–Jensen slants, with pyruvate and without glycerol, were inoculated in parallel and incubated for 6–8 weeks. Suspicious colonies obtained in microbiological cultures were identified as *M. tuberculosis* complex by PCR and “Spoligotyped” following standard methods [25], [26], allowing their identification as *M. bovis*.

In addition, to assess the presence of tuberculosis granulomas, a piece of submandibular or retropharyngeal lymph node and lung of each animal sampled were fixed by immersion in neutral, buffered-formalin (4% formaldehyde) and sections of 4 µm were cut and stained with hematoxylin and eosin for histopathological examination. The tissue pieces used for the histopathological analysis were chosen based on the presence of macroscopic bTB-like lesions when present. For wild boar in which bTB-like lesions were not found, a piece of submandibular lymph node and caudal lung lobe were identically processed for histopathology.

### bTB severity assessment

To consider the extent of bTB, wild boar were classified into two groups: animals with a localized lesion pattern and animals with a generalized lesion pattern. Based on the distribution of lesions, generalized bTB implies more severe disease and a greater bacterial load than localized bTB [27].

**Figure 2:**
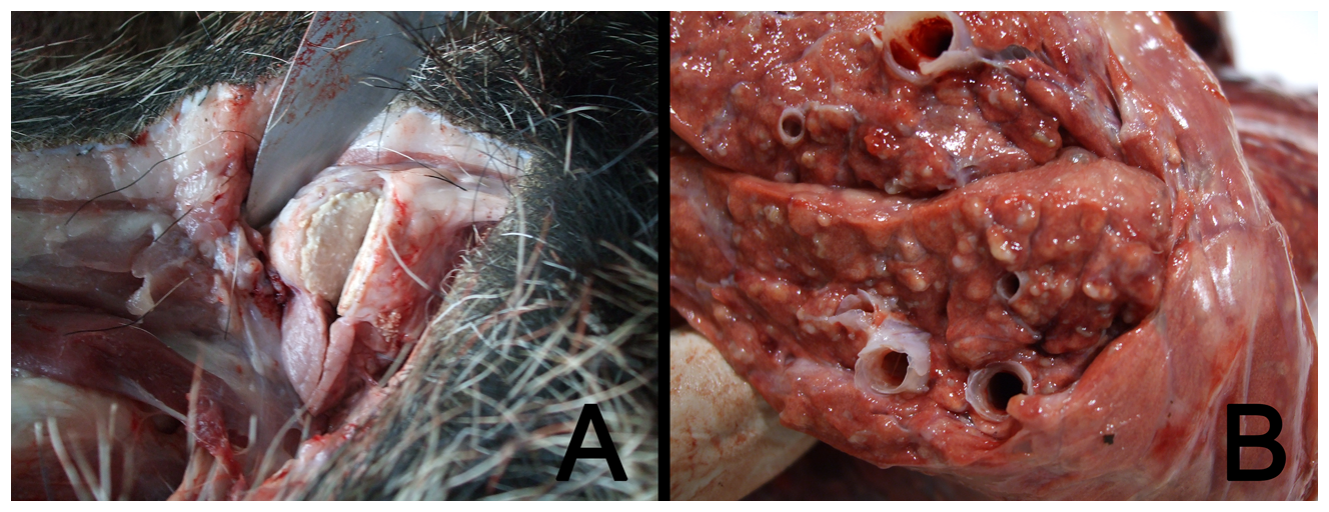
Wild boar showing localized (bTB like lesion in submandibular lymph node) (a) or generalized bTB lesion patterns (bTB like lesions in lungs) (b).

Animals showing a localized pattern were those with bTB lesions in one location, mainly submandibular or retropharyngeal lymph nodes (Figure 2a). On the other hand, those wild boar with lesions in these lymph nodes and any other organ, e.g. lung, liver, mesenteric lymph nodes and/or spleen, were considered to have a generalized pattern (Figure 2b). Since lung is the most common organ in which secondary bTB lesions can be found in wild boar [28], lung tissue was chosen to carry out a systematic detection (through microbiological culture and microscopic examination) of generalized bTB.

A complete lesion assessment could not be carried out in 25 of 165 animals included in this study, since these animals were partially eviscerated upon arrival at the inspection area. This meant we could not ensure the lack of bTB-like lesions in abdominal organs and, therefore, their lesion pattern could not be completely determined.

### Bacterial pathogen/antibody detection

The presence of concomitant bacterial respiratory pathogens, such as *Haemophilus parasuis* and *Actinobacillus pleuropneumoniae*, was determined in lungs of the sampled animals. DNA from a piece of cranial lobe from one of the lungs was extracted using a commercial QIAamp DNA Mini kit (Qiagen Ltd., Crawley, West Sussex, RH10 9NQ, United Kingdom) following the manufacturer's recommendations. Later, specific PCRs were carried out in order to detect the presence of *H. parasuis* [29] and *A. pleuropneumoniae* [30] using previously extracted DNA. Antibodies against *M. hyopneumoniae* were detected using a commercial blocking-ELISA assay for swine (INGEZIM M. HYO COMPAC, Ingenasa, Madrid, Spain) that was carried out using duplicate serum samples from each animal, following the protocol and cut-off values proposed by the manufacturers to differentiate seropositive and seronegative animals (positive threshold  =  sample optical density (OD) <0.40× negative control OD).

### Viral pathogen antibody detection

Concerning viruses, a serologic survey for contacts with viral pathogens was carried out using commercial ELISA kits for swine and also following the manufacturer's recommendations to differentiate seropositive and seronegative animals. The presence of antibodies against PCV2 (INGEZIM CIRCO IgG; positive threshold =  samples OD > negative control OD +0.25), ADV (INGEZIM ADV TOTAL; positive threshold  =  sample OD/positive control OD (S/P) >0.35), SIV (INGEZIM INFLUENZA PORCINA; positive threshold  =  SP >0.2) and PRRSV (INGEZIM PRRS EUROPA; positive threshold =  sample OD > positive control OD ×0.15) was evaluated using duplicate samples of sera obtained from blood samples.

### Metastrongylus spp detection

Pulmonary nematodes were collected by lung dissection. During examination, the trachea and main bronchi were opened longitudinally with scissors, carefully examined and then placed under running water to collect adult worms on a sieve. In addition, the pulmonary parenchyma – and in particular the affected areas – were dissected carefully under a dissecting microscope to extract adult nematodes. Permanent preparations of adult specimens were made with lactophenol cotton blue solution and genus identification was based on previous descriptions [31]. The total number of adult worms collected from an individual [32] was used as a proxy for parasitic load.

A brief summary of the techniques used for antibodies and pathogen detection and the clinical picture produced by bacteria, viruses and parasites assessed in this work are shown in Table 1.

**Table 1:** Brief description of main clinical signs and lesions produced by the respiratory pathogens assessed. The type of assay carried out to diagnose them is also shown.

Pathogen	Type of assay	Clinical signs and lesions	Source
**Bacteria**			
*Mycoplasma hyopneumoniae*	blocking-ELISA	Associated with Enzootic Pneumonia, *M. hyopneumoniae* plays a primary role in the porcine respiratory complex causing important economic losses.	[60]
*Actinobacillus pleuropneumoniae*	PCR	It causes pleuropneumonia that results in death, chronic or subclinical disease causing losses by mortality, reduced production, and increased costs of medication or vaccination.	[61]
*Haemophilus parasuis*	PCR	It is the etiological agent of the porcine arthritis poliserositis (Glasser's Disease) that may produce important losses mainly in intensive farm. Pneumonia in pigs as a primary or secondary agent.	[62]
**Viruses**			
Porcine Circovirus type2	indirect-ELISA	PCV2 infection has been associated with postweaning multisystemic wasting syndrome (PMWS), porcine dermatitis and nephropathy syndrome (PDNS), porcine respiratory disease complex, and reproductive disorders.	[63]
Aujeszky's Disease Virus	indirect-ELISA	May produce high mortality in piglets with neurological disorders. Weaners may show pneumonic symptoms whereas in non-immune sows may produce reproductive disorders.	[64]
Swine Influenza virus	indirect-ELISA	Cause of bronchointerstitial pneumonia and respiratory disease in pigs throughout large parts of the world.	[65]
Porcine Reproductive and Respiratory Syndrome virus	indirect-ELISA	PRRSV infections produce severe reproductive losses, interstitial pneumonia in pigs, reduction of growth performance, and increased mortality.	[66]
***Helminths***			
*Metastrongylus spp*	Direct retrieval at necropsy	It produces chronic granulomatous pneumonic lesions mainly in caudal lobes of the lungs. Cough with minimal other signs.	[67]

### Statistical Procedures

For the analyses described below, all estates showed the presence of the most prevalent pathogens, e.g., *M. bovis*, PCV2, ADV, SIV and *Metastrongylus* spp. Neither differences in pathogen prevalences, pathogen community structure nor in the effect of co-infection on bTB severity were determined at the game estate scale, in part because the sample size never exceeded 30 individuals per estate.

#### Pathogen community

Whether or not contact with other pathogens in wild boar infected by *Mycobacterium* spp occurred by chance was studied using the null model analysis. Data were organised as presence/absence matrices in which each row represented a pathogen species and each column represented an individual wild boar. In a presence/absence matrix, “1” indicates that a species is present at a particular site or host, and “0” indicates that a species is absent [33]. A total of three matrices were created separately for each age class: juveniles (6–12 months), yearlings (13–24 months) and adults (over 24 months).

The C-score was used as a co-occurrence index for exploring co-occurrence patterns [34] and the FE algorithm (fixed row-equiprobable column) chosen to analyze the results obtained. The C-score measures the average number of checkerboard units between all possible pairs of species. The C-score measures the average number of checkerboard units between all possible pairs of species. In a competitively structured community, the observed C-score should be significantly larger than expected by chance (O >E). Otherwise, a C-score smaller than expected by chance (O<E) indicates a randomly assembled community [35], i.e. a greater likelihood that the distribution of one species has been directly affected by the presence of other species. The C-score has been used in diverse null models as a powerful tool to measure not only parasite assemblages [36], but also viral co-infections in pigs [37]. The observed C-score was calculated for each presence/absence matrix and compared with the expected C-score calculated for 5000 randomly assembled null matrices by Monte Carlo procedures. The analysis was carried out using the software EcoSim 7.72 [38].

In addition, to compare the degree of co-occurrence across data, a standardised effect size (SES) for each matrix was calculated. The SES measures the number of standard deviations that the observed index (C-score) is above or below the mean index of the simulated communities.

#### The role of co-infection in bTB severity

The association between viruses, bacteria and nematode species (by means of direct detection or serological evidence of infection) and bTB severity (0 for wild boar showing bTB-like lesions only in submandibular/retropharyngeal lymph nodes, and 1 for those showing bTB-like lesions in both lymph nodes and lungs) was assessed by a Partial Least Squares regression approach (PLSr). This statistical tool is an extension of multiple regression analysis, where associations between groups of variables are established with factors, i.e., combinations of dependent variables extracted from predictor variables that maximise the explained variance in the dependent variables. PLSr is probably the least restrictive of the multivariate techniques [39]. This flexibility allows its use when there are fewer observations than predictor variables or in the case of multicollinearity [40]. Moreover, PLSr allows the study of covariance in both explanatory and predictor variable groups [41].

In the present study, bTB severity was considered as a single explanatory variable, while concomitant pathogens represented the × predictor factor. For PLSr modelling, the age of animals was considered in months. The “plspm” library version 0.3.7 [42] of the R software version 3. 0. 3 [43] was used for these analyses.

## Results

### Percentage of animals positive against selected pathogens

The *M. bovis* isolates were obtained from 85 animals (51.51%). Within infected animals, 28 showed *M. bovis* infection in both submandibular/retropharyngeal lymph nodes and lungs (generalized pattern), whereas 57 only showed *M. bovis* infection in submandibular/retropharyngeal lymph nodes (localized pattern). On the other hand, bTB-like microscopic lesions were observed in 80 submandibular/retropharyngeal lymph nodes (48.48%, n = 165 lymph nodes) and 28 lungs (16.96%, n = 165 lungs). Nine out of 85 animals infected by *M. bovis* (10.58%) did not show evidence of microscopic bTB-like lesions, while this microorganism could not be isolated from four animals that showed typical bTB granulomatous lesions in their lymph nodes (5%). Thus, since the combination of microbiological culture and histopathology increases the sensitivity of bTB diagnosis in wild boar [23], these 89 animals were considered positive to bTB (53.94%). All the isolates obtained were identified as *M. bovis* and showed 12 different spoligotype patterns (SB0119 (15.29%), SB0121 (25.88%), SB0134 (4.71%), SB0296 (3.53%), SB0339 (23.53%), SB1091 (8.23%), SB1142 (8.23%), SB0120 (1.18%), SB0152 (3.53%), SB0848 (3.53%), SB1142 (8.23%), SB1174 (2.35%)).

A detailed lesion severity assessment was carried out in 71 out of 89 animals positive to bTB. Generalized lesion patterns were detected in 28 bTB affected animals (40%), whereas localized lesions were observed in 43 affected animals (60%). Some of the animals with generalized patterns also displayed gross bTB-like lesions in organs such as liver, spleen or mesenteric lymph nodes; however, no bTB-like lesions were found in these organs in animals that did not show bTB-like lesions in the lungs (see (Table S1)).

The percentage of animals positive for selected respiratory pathogens is shown in Table 2. High rates of seropositive animals were found against PCV2 (70.9%) and ADV (69.7%), while lower percentages were found against other pathogens such as SIV (24.24%) and *M. hyopneumoniae* (13.94%). *A. pleuropneumoniae* and *Metastrongylus* spp were detected in 4.84% and 50.51% of the animals, respectively, whereas evidence of infections with PRRSV or *H. parasuis* was not detected.

**Table 2:** Percentage of animals positive against the selected respiratory pathogens included in this study in 24 juveniles (6–12 months), 45 yearlings (13–24 months) and 96 adult (over 24 months) wild boar hunter harvested in mid-western Spain.

Pathogens	Percentage of positive animals	Age-specific percentage of positive animals
***Virus***		
Porcine circovirus type 2	70.9%	Juveniles: 58.33%
		Yearlings: 71.11%
		Adults: 73.96%
Aujeszky's disease virus	69.70%	Juveniles: 45.83%
		Yearlings: 55.56%
		Adults: 82.80%
Swine influenza virus	24.24%	Juveniles: 16.67%
		Yearlings: 13.33%
		Adults: 31.25%
PRRS virus	0%	Juveniles: 0%
		Yearlings: 0%
		Adults: 0%
***Bacteria***		
*Mycobacterium bovis*	53.93%	Juveniles: 54.16%
		Yearlings: 57.78%
		Adults: 52.08%
*Haemophilus parasuis*	0%	Juveniles: 0%
		Yearlings: 0%
		Adults: 0%
*Actinobacillus pleuropneumoniae*	4.84%	Juveniles: 0%
		Yearlings: 4.44%
		Adults: 6.25%
*Mycoplasma hyopneumoniae*	13.94%	Juveniles: 16.67%
		Yearlings: 17.78%
		Adults: 11.46%
***Helminths***		
*Metastrongylus spp*	51.51%	Juveniles: 66.67%
		Yearlings: 57.78%
		Adults: 44.79%

### Pathogen community structure

The most common helminth, bacteria and viruses assemblages observed in *M. bovis* infected juvenile, yearling and adult wild boar are shown in Table 3. Wild boar with negative results for all the pathogens tested were rare (5.5% of cases) as were wild boar infected with bTB and all the other pathogens studied (just one individual, Table 3). The 29.6% of adults, 14.5% of yearlings and 25.1% of juveniles that were *M. bovis* infected showed antibodies against PCV2 and ADV.

**Table 3:** Observed frequencies for different pathogen assemblages (%) including *Metastrongylus* spp and *Mycobacterium bovis* infection and, *Mycoplasma hyopneumoniae* (Mhyo), Porcine circovirus type 2 (PCV2), Swine influenza virus (SIV), and Aujeszky's disease virus (ADV) antibodies positivity in 165 adult, yearling and juvenile males and females wild boar hunter-harvested in mid-western Spain.

Pathogen group						Age class		
Helminths	Bacteria		Virus			Percentage		
*Metastrongylus* spp	*M. bovis*	*Mhyo*	PCV2	SIV	ADV	Adults	Yearlings	Juveniles
0	1	0	1	0	1	**11.2%**	3.6%	4.2%
1	1	0	1	0	1	**9.2%**	**9.1%**	**16.7%**
1	1	0	1	1	1	**9.2%**	1.8%	4.2%
0	0	0	1	0	1	**9.2%**	**7.3%**	0%
1	0	0	1	0	1	**8.2%**	**7.3%**	**8.3%**
1	1	0	1	0	0	2%	**9.1%**	**8.3%**
0	0	0	1	0	0	3.1%	**7.3%**	4.2%
0	1	0	1	0	0	3.1%	**7.3%**	4.2%
0	0	1	1	0	0	0%	**7.3%**	0%
0	0	0	0	0	0	2%	**5.5%**	4.2%
1	0	0	0	0	1	2%	**5.5%**	4.2%
0	0	0	1	0	0	3.1%	**7.3%**	4.2%
1	1	1	1	1	1	1%	0%	0%

Zero indicates lack of detectable antibodies or pathogen whereas 1 indicates presence of antibodies against the specific pathogen. For *Metastrongylus* spp and *M. bovis*, 1 indicates presence of the pathogen in the corresponding samples. In bold, some specific combinations that appeared in more than 5% of studied boars.

The null model analysis showed that the observed C-scores were greater than expected by chance (O >E) indicating the existence of a competitively structured community; that is, bTB-infected wild boar have contacted with some specific, non-random pathogen combinations (Table 3). This fact was especially evident for adult animals, which showed the only statistically significant result (Table 4).

**Table 4:** Observed (O) and expected by chance (E) values of the C-score for positive/negative matrices of virus, bacteria and helminth communities on 24 juveniles, 45 yearlings and 96 adult wild boar from mid-western Spain.

Age class	C-score			
	O	E	p	SES
Juveniles	4.46	4.19	0.15	1.14
Yearlings	19.31	18.70	0.78	0.68
Adults	39.93	36.11	0.01	2.71

### Effects of co-infection on bTB severity

**Figure 3:**
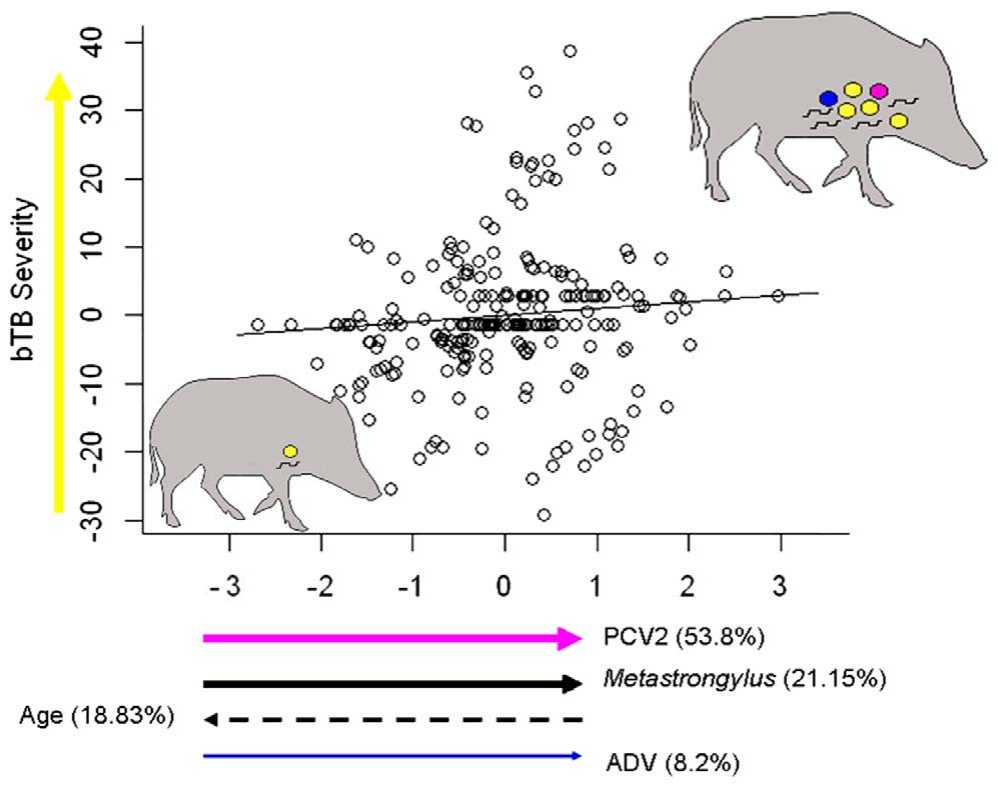
Relationships between exposure to viral infections (PCV2, arrow in pink, and ADV, in blue), nematode infection (arrow in black) and age on PLSr component describing disease severity (arrow in yellow) in *Mycobacterium bovis* infected wild boar. This plot represents the PLSr model shown in Table 5. Arrow way indicates either an increase or decrease in the component value. Arrow thickness directly indicates the contribution of each variable to PLSr X's component. Since swine influenza virus and *Mycoplasma hyopneumoniae* explained less than 10% of the PLSr X's component, they were not represented in this plot. The wild boar silhouettes summarize those pathogen combinations linked to severe bovine tuberculosis (represented by several circles in yellow). The more yellow circles a wild boar has, the more severe is the disease. The rest of coloured circles represent co-infections by different pathogens. Each colour matches with species represented by the arrows (i.e., pink for PCV2 or blue for ADV). Nematodes, however, are represented by the black short lines within the silhouettes.

In the PLSr analysis, presence of PCV2, SIV and ADV antibodies, *Metastrongylus* spp, sex and age provided a first PLSr X's component explaining 20.90% of the observed variability (Table 5). More than 90% of the total variance explained by the PLSr × axis was due not only to virus exposure (PCV2 and ADV) but also to age of animals and infection by lung nematodes. The weights of variables performing the explanatory X's component describing the severity of bTB infection had different signs. A positive correlation of bTB infection severity was found with evidence of PCV2 and ADV contact and presence of *Metastrongylus* spp, whereas a negative association was related to age (Figure 3). Finally, the sex of animals and the presence of antibodies against SIV and *Mycoplasma hyopneumoniae*, appeared to have a lower influence on tuberculosis severity.

**Table 5:** Predictor weights of the Partial Least Squares regression (PLSr) model explaining the effects of presence of antibodies elicited by porcine circovirus type 2 (PCV2), Aujeszky's disease virus (ADV), swine influenza virus (SIV), *Mycoplasma hyopneumoniae* and co-infection by *Metastrongylus* lung nematodes on bTB severity.

Pathogens	Predictor weights	% Variance explained
**PCV2**	0.676	53.80
***Metastrongylus*** ** spp**	0.469	21.15
**Age**	−0.405	18.83
**ADV**	0.318	8.2
SIV	−0.228	6.31
Sex	−0.078	0.77
*Mycoplasma hyopneumoniae*	0.011	0.05

Predictor weights represent the contribution of each pathogen infection to the PLSr's × axis. Predictor weights explaining more than 10% of the total variance in each response variable are shown in bold type. The exposure to porcine reproductive and respiratory syndrome virus (PRRSV), *Haemophilus parasuis*, and *Actinobacillus pleuropneumoniae* were excluded from this analysis since their prevalence in studied wild boar population was lower than 5% (see table 2).

## Discussion

Results obtained in this work suggest that evidences of infection with common wild boar pathogens such as PCV2, *Metastrongylus* spp and ADV are associated with a higher likelihood of detecting generalized bTB lesions. This has already been observed in animal models co-infected by two pathogens [17], [44]. However, to date no work has evaluated the relationship between a more complex pathogen community and bTB severity. Current results emphasize the importance of considering a broad representation of the pathogen community to assess the existence of possible associations between them [45].

Our PLSr modelling including “age” and “contact with PCV2, ADV or *Metastrongylus* spp” as risk factors explained more than 20% of the likelihood of showing generalized bTB lesion patterns in wild boar. Although the percentage of explained variance of the observed variability in bTB disease severity can be considered adequate (note that r between 0.14 to 0.25 are the most common effect sizes in observational ecological works [46]), other factors not included in this study might also be associated with the presence of bTB severe patterns in wild boar. Pathogens evaluated in the present work, including viruses, bacteria and helminths, represent a broad range of mainly respiratory wild boar pathogens. However, other pathogens like gastro-intestinal nematodes, which influence the development of bTB in other wildlife species [17] may also play a role. In addition, other factors such as infective dose of *M. bovis* or host genetics may also influence the development of bTB in wild boar [47] and, hence, future studies taking into account these variables might complete the results obtained in the present work.

Regarding the order and timing of infections, the current study does not allow the determination of whether contact with these other pathogens was previous, simultaneous or subsequent to the precise time of infection by *M. bovis* in the studied animals. However, co-infection with other pathogens may affect the severity of tuberculosis in all of these temporal situations, by reactivating the infection in animals previously infected with *Mycobacterium* spp. (leading to more severe tuberculosis) [48] or by allowing a more rapid expansion of the lesions (increasing the severity) in animals that were infected with *Mycobacterium* spp. later [49].

Diagnosis of some of the pathogens assessed in this work was based on serological techniques. Detection of antibodies against one pathogen does not necessarily mean a current infection since high titers of antibodies may remain for a long time after the clearance of the agent. However, this limitation might not influence the results obtained in this study since bTB is a chronic disease and lesions may persist in an affected wild boar for a long time [50]. Thus, serological analyses allow the exploration of the possible association between past or current co-infections and the severity of bTB, defined by the detection of a generalized pattern of lesions at the moment of sampling.

Differentiation between seropositive and seronegative animals was carried out using the cut off values recommended by the ELISA's manufacturers, as has been previously done in similar surveys carried out in wild boar [51]–[53]. To date, sensitivity and specificity values of these tests in wild boar have not been provided by the manufacturers or by any study, and hence, we could not estimate the influence of these parameters in the seroprevalences obtained.

According to our results, certain pathogen combinations are correlated to more severe bTB lesions in wild boar. Since the presence of animals displaying severe bTB lesion patterns has been recently related to higher bTB prevalence in wild boar populations [19], programs aimed at bTB management and control in wild boar populations should take into account the effects of concomitant pathogens on the disease severity. The relationship between PCV2, *Metastrongylus* spp and ADV on bTB severity suggests that sanitary measures focused on the control of these agents (i.e., deworming or vaccination against viruses), may help to reduce the bTB prevalence in the affected areas.

Importantly, the interaction between the above-mentioned pathogens and *M. bovis* may influence the success of measures applied to reduce bTB prevalence in wild boar populations, such as bTB vaccination [54]. It has been shown that the protective efficacy of bTB vaccination in animals previously infected by helminths is deficient [44]. The biased Th2 immune response and hyporesponsiveness associated with chronic helminthiases might impair their ability to mount an effective immune response after vaccination [55]. Thus, the presence of *Metastrongylus* spp in wild boar vaccinated against bTB may reduce the effectiveness of this vaccine whereas measures such as previous deworming may be useful in the estates where vaccination will be carried out. In fact, a significant improvement in mycobacterial-specific immune responses occurs following anthelmintic therapy in vaccinated humans [56]. However, experiences of deworming for disease control in both humans and wild animals have provided very contradictory results [57]. Consequently, these types of alternative management measures might have different consequences for disease control and should be carefully evaluated under different situations before any generalisation.

On the other hand, it is noteworthy that the greatest effects of co-infections in other wild hosts have been observed in seasons of food shortage [58]. In this sense, measures focused on maintaining a good nutritional status in wild boar would be essential for reducing the negative effects of concomitant pathogens in the development of bTB in this species, hence helping to reduce the prevalence of bTB in wild boar. In any case, further investigations are required before a massive supplemental feeding aimed at bTB control is used in wild boar populations, since artificial feeding favours an increased helminth infection rate in wild boar [59]. In addition, supplemental feeding could be beneficial mainly in fenced estates with artificially high densities, because it can increase wild boar densities in natural populations and might raise the risk of transmission of *M. bovis* in these populations.

To conclude, results obtained in this work provide a new point of view for bTB control based on community ecology principles. Removing specific members of the wild boar pathogen community could be considered in further bTB management plans in wild boar. However, given the unexpected consequences of altering any community of organisms, further research should evaluate the impact of such measures under controlled conditions. Furthermore, agents assessed in this work represent a limited group of wild boar pathogens. Therefore, the influence that other agents (eg. gastrointestinal nematodes) have in wild boar bTB severity should be explored in future studies to obtain a more complete picture.

## Supporting Information

Table S1 **Results obtained in characterisation, laboratory diagnosis and lesional assessment carried out in the 165 wild boar included in the study.**
(XLSX)

## References

[1] Pérez JM, Meneguz PG, Dematteis A, Rossi L, Serrano E (2006) Parasites and conservation biology: The 'ibex-ecosystem'. Biodivers Conserv 15: 2033–2047.

[2] Hatcher MJ, Dunn AM (2011) Parasites in ecological communities, from interactions to ecosystems. Cambridge: Cambridge University Press. 439 p.

[3] Reich NG, Shrestha S, King AA, Rohani P, Lessler J, et al (2013) Interactions between serotypes of dengue highlight epidemiological impact of cross-immunity. J R Soc Interface 10: 86. 10.1098/rsif.2013.0414 PMC3730691 23825116

[4] Telfer S, Lambin X, Birtles R, Beldomenico P, Burthe S, et al (2010) Species interactions in a parasite community drive infection risk in a wildlife population. Science 330: 243–246. 20929776 10.1126/science.1190333 PMC3033556

[5] Yacob HT, Terefe G, Jacquiet P, Hoste H, Grisez C, et al (2006) Experimental concurrent infection of sheep with *Oestrus ovis* and *Trichostrongylus colubriformis*: Effects of antiparasitic treatments on interactions between parasite populations and blood eosinophilic responses. Vet Parasitol 137: 184–188. 16487660 10.1016/j.vetpar.2006.01.020

[6] Graham AL, Cattadori IM, Lloyd-Smith JO, Ferrari MJ, Bjornstad ON (2007) Transmission consequences of coinfection: cytokines writ large? Trends Parasitol 23: 284–291. 17466597 10.1016/j.pt.2007.04.005

[7] Supali T, Verweij JJ, Wiria AE, Djuardi Y, Hamid F, et al (2010) Polyparasitism and its impact on the immune system. Int J Parasitol 40: 1171–1176. 20580905 10.1016/j.ijpara.2010.05.003

[8] Pathak AK, Pelensky C, Boag B, Cattadori IM (2012) Immuno-epidemiology of chronic bacterial and helminth co-infections: Observations from the field and evidence from the laboratory. Int J Parasitol 42: 647–655. 22584129 10.1016/j.ijpara.2012.04.011

[9] Lass S, Hudson PJ, Thakar J, Saric J, Harvill E, et al (2013) Generating super-shedders: Co-infection increases bacterial load and egg production of a gastrointestinal helminth. J R Soc Interface 10: 80. 10.1098/rsif.2012.0588 PMC3565725 23256186

[10] Thakar J, Pathak AK, Murphy L, Albert R, Cattadori IM (2012) Network model of immune responses reveals key effectors to single and co-infection dynamics by a respiratory bacterium and a gastrointestinal helminth. PLoS Comp Biol 8: e1002345. 10.1371/journal.pcbi.1002345 PMC3257297 22253585

[11] Black TC (1946) Coexistent hookworm and tuberculosis. South Med J 39: 881–884. 21002667 10.1097/00007611-194611000-00007

[12] Li XX, Zhou XN (2013) Co-infection of tuberculosis and parasitic diseases in humans: A systematic review. Parasit Vectors 6: 79. 23522098 10.1186/1756-3305-6-79 PMC3614457

[13] Pawlowski A, Jansson M, Sköld M, Rottenberg ME, Källenius G (2012) Tuberculosis and HIV co-infection. PLoS Path 8: e1002464. 10.1371/journal.ppat.1002464 PMC3280977 22363214

[14] Bordes F, Morand S (2011) The impact of multiple infections on wild animal hosts: a review. Infect Ecol Epidemiol 1: 7346. 10.3402/iee.v1i0.7346 PMC3426331 22957114

[15] Fitzgerald SD, Kaneene JB (2013) Wildlife Reservoirs of Bovine Tuberculosis Worldwide: Hosts, Pathology, Surveillance, and Control. Vet Pathol 50: 488–499. 23169912 10.1177/0300985812467472

[16] Gortazar C, Cowan P (2013) Introduction to this issue: Dealing with TB in wildlife. Epidemiol Infect 141: 1339–1341. 23894746 10.1017/S0950268813000599 PMC9151608

[17] Ezenwa VO, Etienne RS, Luikart G, Beja-Pereira A, Jolles AE (2010) Hidden consequences of living in a wormy world: Nematode-induced immune suppression facilitates tuberculosis invasion in African buffalo. Am Nat 176: 613–624. 20849271 10.1086/656496

[18] Jolles AE, Ezenwa VO, Etienne RS, Turner WC, Olff H (2008) Interactions between macroparasites and microparasites drive infection patterns in free-ranging African buffalo. Ecology 89: 2239–2250. 18724734 10.1890/07-0995.1

[19] Risco D, Fernández-Llario P, García-Jimenez WL, Gonçalves P, Cuesta JM, et al (2013) Influence of porcine circovirus type 2 infections on bovine tuberculosis in wild boar populations. Trans Emerg Dis 60 Suppl 1 121–127. 10.1111/tbed.12112 24171857

[20] Rafi W, Ribeiro-Rodrigues R, Ellner JJ, Salgame P (2012) 'Coinfection-helminthes and tuberculosis'. Curr Opin HIV AIDS 7: 239–244. 22411453 10.1097/COH.0b013e3283524dc5

[21] Risco D, García A, Serrano E, Fernández-Llario P, Benítez JM, et al. (2013) High-Density Dependence But Low Impact on Selected Reproduction Parameters of Brucella suis Biovar 2 in Wild Boar Hunting Estates from South-Western Spain. Trans Emerg Dis: In press. 10.1111/tbed.12060 23347330

[22] Boitani L, Mattei L (1992) Aging wild boar (*Sus scrofa*) by tooth eruption. In: Spitz F, Janeau G, González G, Aulagnier S, editors. Ongules/Ungulates 91. Tolouse: SFEPM-IRGM. pp. 419–421.

[23] Santos N, Geraldes M, Afonso A, Almeida V, Correia-Neves M (2010) Diagnosis of Tuberculosis in the Wild Boar (*Sus scrofa*): A comparison of Methods Applicable to Hunter-Harvested Animals. PLoS ONE 5: e12663. 20844754 10.1371/journal.pone.0012663 PMC2937024

[24] Corner LA, Trajstman AC (1988) An evaluation of 1-hexadecylpyridinium chloride as a decontaminant in the primary isolation of *Mycobacterium bovis* from bovine lesions. Vet Microbiol 18: 127–134. 3064398 10.1016/0378-1135(88)90058-2

[25] Cousins DV, Wilton SD, Francis BR (1991) Use of DNA amplification for the rapid identification of *Mycobacterium bovis* . Vet Microbiol 27: 187–195. 2063549 10.1016/0378-1135(91)90010-d

[26] Kamerbeek J, Schouls J, Kolk A, van Agterveld M, van Soolingen D, et al (1997) Simultaneous detection and strain differentiation of *Mycobacterium tuberculosis* for diagnosis and epidemiology. J Clin Microbiol 35: 907–914. 9157152 10.1128/jcm.35.4.907-914.1997 PMC229700

[27] Menin Ã, Fleith R, Reck C, Marlow M, Fernandes P, et al (2013) Asymptomatic Cattle Naturally Infected with *Mycobacterium bovis* Present Exacerbated Tissue Pathology and Bacterial Dissemination. PLoS ONE 8: e53884. 23326525 10.1371/journal.pone.0053884 PMC3541226

[28] Martín-Hernando MP, Höfle U, Vicente J, Ruiz-Fons F, Vidal D, et al (2007) Lesions associated with Mycobacterium tuberculosis complex infection in the European wild boar. Tuberculosis 87: 360–367. 17395539 10.1016/j.tube.2007.02.003

[29] Oliveira S, Galina L, Pijoan C (2001) Development of a PCR test to diagnose *Haemophilus parasuis* infections. J Vet Diagn Investig 13: 495–501. 11724140 10.1177/104063870101300607

[30] Cho WS, Chae C (2003) PCR detection of *Actinobacillus pleuropneumoniae* apxIV gene in formalin-fixed, paraffin-embedded lung tissues and comparison with in situ hybridization. Lett Appl Microbiol 37: 56–60. 12803557 10.1046/j.1472-765x.2003.01347.x

[31] Anderson DR, Burnham KP, Thompson WL (2000) Null hypothesis testing: Problems, prevalence, and an alternative. J Wildl Manage 64: 912–923.

[32] Bush AO, Lafferty KD, Lotz JM, Shostak AW (1997) Parasitology meets ecology on its own terms: Margolis, et al. revisited. Parasitolgy 83: 575–583. 9267395

[33] Gotelli NJ (2000) Null model analysis of species co-occurrence patterns. Ecology 81: 2606–2621.

[34] Stone L, Roberts A (1990) The checkerboard score and species distributions. Oecologia 85: 74–79. 28310957 10.1007/BF00317345

[35] Gotelli NJ, McCabe DJ (2002) Species co-occurrence: A meta-analysis of J. M. Diamond's assembly rules model. Ecology 83: 2091–2096.

[36] Gotelli NJ, Rohde K (2002) Co-occurrence of ectoparasites of marine fishes: A null model analysis. Ecol Lett 5: 86–94.

[37] Serrano E, López-Soria S, Trinchera L, Segalés J (2014) The use of null models and partial least squares approach path modelling (PLS-PM) for investigating risk factors influencing post-weaning mortality in indoor pig farms. Epidemiol Infect 142: 530–539. 23725592 10.1017/S0950268813001295 PMC9151122

[38] Calero-Bernal R, Gómez-Gordo L, Saugar JM, Frontera E, Pérez-Martín JE, et al (2013) Congenital toxoplasmosis in wild boar (*Sus scrofa*) and identification of the *Toxoplasma gondii* types involved. J Wildl Dis 49: 1019–1023. 24502733 10.7589/2013-01-024

[39] Haenlein M, Kaplan AM (2004) A beginners's guide to Partial Least Squares analysis. Underst Stat 3: 283–297.

[40] Geladi P, Kowalski B (1986) Partial Least Squares Regression: a tutorial. Anal Chim Acta 185: 1–17.

[41] Abbi H (2007) Partial least square regression PLS-Regression. In: Salkind N, editor. Encyclopedia of Measurement and Statistics: Thousans Oaks. pp. 741–745.

[42] Sánchez G, Trinchera L (2010) plspm: Partial Least Squares data analysis methods. R package version 0 3 7

[43] Team RDC (2014) R: A language and environment for statistical computing. Vienna: R Foundation for Statistical Computing.

[44] Elias D, Akuffo H, Pawlowski A, Haile M, Schön T, et al (2005) *Schistosoma mansoni* infection reduces the protective efficacy of BCG vaccination against virulent *Mycobacterium tuberculosis* . Vaccine 23: 1326–1334. 15661380 10.1016/j.vaccine.2004.09.038

[45] Serrano E, Millán J (2013) What is the price of neglecting parasite groups when assessing the cost of co-infection? Epidemiol Infect 142: 1533–1540. 24040768 10.1017/S0950268813002100 PMC9151198

[46] Moller AP, Jennions MD (2002) How much variance can be explained by ecologist and evolutionary biologists? Oecologia (Berl) 132: 492–500. 28547634 10.1007/s00442-002-0952-2

[47] Acevedo-Whitehouse K, Vicente J, Gortázar C, Höfle U, Fernández-De-Mera IG, et al (2005) Genetic resistance to bovine tuberculosis in the Iberian wild boar. Mol Ecol 14: 3209–3217. 16101786 10.1111/j.1365-294X.2005.02656.x

[48] Diedrich CR, Flynn JL (2011) HIV-1/*Mycobacterium tuberculosis* coinfection immunology: How does HIV-1 exacerbate tuberculosis? Infect Immun 79: 1407–1417. 21245275 10.1128/IAI.01126-10 PMC3067569

[49] Elias D, Akuffo H, Thors C, Pawlowski A, Britton S (2005) Low dose chronic *Schistosoma mansoni* infection increases susceptibility to *Mycobacterium bovis* BCG infection in mice. Clin Exp Immunol 139: 398–404. 15730384 10.1111/j.1365-2249.2004.02719.x PMC1809318

[50] Bollo E, Ferroglio E, Dini V, Mignome W, Biolatti B, et al (2000) Detection of *Mycobacterium tuberculosis* Complex in Lymph Nodes of Wild Boar (*Sus scrofa*) by a Target-Amplified Test System. J Vet Med B/Zentralbl Veterinaermed Reihe B 47: 337–342. 10.1046/j.1439-0450.2000.00354.x 10900824

[51] Hammer R, Ritzmann M, Palzer A, Lang C, Hammer B, et al (2012) Porcine Reproductive and Respiratory Syndrome Virus and Porcine Circovirus Type 2 Infections in Wild Boar (Sus Scrofa) in Southwestern Germany. J Wildl Dis 48: 87–094. 22247377 10.7589/0090-3558-48.1.87

[52] Štukelj M, Toplak I, Vengušt G (2014) Prevalence of antibodies against selected pathogens in wild boars (Sus scrofa) in Slovenia. Slovenian Veterinary Research 51: 21–28.

[53] Roic B, Jemersic L, Terzic S, Keros T, Balatinec J, et al (2012) Prevalence of antibodies to selected viral pathogens in wild boars (Sus scrofa) in Croatia in 2005–06 and 2009–10. J Wildl Dis 48: 131–137. 22247381 10.7589/0090-3558-48.1.131

[54] Garrido JM, Sevilla IA, Beltrán-Beck B, Minguijón E, Ballesteros C, et al (2011) Protection against tuberculosis in Eurasian wild boar vaccinated with heat-inactivated Mycobacterium bovis. PLoS ONE 6: e24905. 21935486 10.1371/journal.pone.0024905 PMC3173485

[55] Borkow G, Bentwich Z (2004) Chronic immune activation associated with chronic helminthic and human immunodeficiency virus infections: Role of hyporesponsiveness and anergy. Clin Microbiol Rev 17: 1012–1030. 15489359 10.1128/CMR.17.4.1012-1030.2004 PMC523563

[56] Elias D, Wolday D, Akuffo H, Petros B, Bronner U, et al (2001) Effect of deworming on human T cell responses to mycobacterial antigens in helminth-exposed individuals before and after bacille Calmette-Guérin (BCG) vaccination. Clin Exp Immunol 123: 219–225. 11207651 10.1046/j.1365-2249.2001.01446.x PMC1905995

[57] Fenton A (2013) Dances with worms: the ecological and evolutionary impacts of deworming on coinfecting pathogens. Parasitology 140: 1119–1132. 23714427 10.1017/S0031182013000590 PMC3695730

[58] Ezenwa VO, Jolles AE (2011) From host immunity to pathogen invasion: The effects of helminth coinfection on the dynamics of microparasites. Integr Comp Biol 51: 540–551. 21727178 10.1093/icb/icr058

[59] Navarro-González N, Fernández-Llario P, Pérez E, Mentaberre G, Lavín S, et al (2013) Supplemental feeding drives parasite comunities of wild boar in Western Spain. Vet Parasitol 196: 114–123. 23537946 10.1016/j.vetpar.2013.02.019

[60] Thacker EL (2006) Mycoplasmal Diseases. In: Straw B, Zimmermann J, D'Allaire S, Taylor DJ, editors. Diseases of Swine. 9th ed. Ames, Iowa: Blackwell Publishing. pp. 701–718.

[61] Gottschalk M, Taylor DJ (2006) Actinobacillus pleuropneumoniae. In: Straw B, Zimmermann J, D'Allaire S, Taylor DJ, editors. Diseases of Swine. 9th ed. Ames, Iowa: Blackwell Publishing. pp. 563–576.

[62] Rapp-Gabrielson VJ, Oliveira SR, Pijoan C (2006) Haemophilus parasuis. In: Straw B, Zimmermann J, D'Allaire S, Taylor DJ, editors. Diseases of Swine. 9th ed. Ames, Iowa: Blackwell Publishing. pp. 681–690.

[63] Segalés J, Allan GM, Domingo M (2006) Porcine Circovirus Diseases. In: Straw B, Zimmermann J, D'Allaire S, Taylor DJ, editors. Diseases of Swine. 9th ed. Ames, Iowa: Blackwell Publishing. pp. 299–309.

[64] Pejsak ZK, Truszczynsky MJ (2006) Aujeszky's Disease. In: Straw B, Zimmermann J, D'Allaire S, Taylor DJ, editors. Diseases of Swine. 9th ed. Ames, Iowa: Blackwell Publishing. pp. 419–435.

[65] Olsen CW, Brown IH, Easterday BC, van Reeth K (2006) Swine Influenza. In: Straw B, Zimmermann J, D'Allaire S, Taylor DJ, editors. Diseases of Swine. 9th ed. Ames, Iowa: Blackwell Publishing. pp. 469–483.

[66] Zimmerman J, Benfield DA, Murtaugh MP, Osorio F, Stevenson GW, et al. (2006) Porcine Reproductive and Respiratory Syndrome Virus (Porcine Arterivirus). In: Straw B, Zimmermann J, D'Allaire S, Taylor DJ, editors. Diseases of Swine. 9th ed. Ames, Iowa: Blackwell Publishing. pp. 387–419.

[67] García-González ÁM, Pérez-Martín JE, Gamito-Santos JA, Calero-Bernal R, Alcaide Alonso M, et al (2013) Epidemiologic study of lung parasites (*Metastrongylus* spp.) in wild boar (*Sus scrofa*) in southwestern Spain. J Wildl Dis 49: 157–162. 23307382 10.7589/2011-07-217

